# Tumor proportion in colon cancer: results from a semiautomatic image analysis approach

**DOI:** 10.1007/s00428-020-02764-1

**Published:** 2020-02-19

**Authors:** Benedikt Martin, Bettina Monika Banner, Eva-Maria Schäfer, Patrick Mayr, Matthias Anthuber, Gerhard Schenkirsch, Bruno Märkl

**Affiliations:** 1 Institute of Pathology and Molecular Diagnostics, University Hospital Augsburg, Augsburg, Germany; 2 Department of Radiooncology, University Hospital Augsburg, Augsburg, Germany; 3 Department of Visceral Surgery, University Hospital Augsburg, Augsburg, Germany; 4 Tumor Data Management, University Hospital Augsburg, Augsburg, Germany

**Keywords:** Colon cancer, Tumor stroma ratio, Biomarker, ImageJ

## Abstract

The tumor stroma ratio (TSR) is a promising prognostic biomarker in colon cancer, which could provide additional risk stratification for therapy adaption. The objective of our study was the investigation of the prognostic significance of TSR at different tumor sites in a simple semiautomatic approach with the open-source program ImageJ. We investigated 206 pT3 and pT4 adenocarcinomas of no special type. According to our established thresholds, 31 tumors (15%) were classified as low tumor proportion (TP) (≤ 15% TP), 42 tumors (20%) were classified as high TP (≥ 54% TP), and 133 tumors (65%) were classified as medium TP. High and low TP were associated with an adverse overall survival in comparison to medium TP (*p* = 0.001 and *p* = 0.03). Furthermore, the TP was an independent risk factor of occurrence of distant metastasis next to T status, microsatellite status, and tumor budding. The 5-year survival rate was 49% in patients with high TP, 48% in patients with low TP, and 68% in patients with medium TP (*p* = 0.042, *n* = 160). Patients with a high TP had less often tumor budding (*p* = 0.012), lymphovascular invasion (*p* = 0.049), and less harvested lymph nodes (*p* = 0.042) in comparison to low TP tumors. The results provide first evidence that a high tumor proportion/low stroma proportion is also associated with an adverse prognosis and that this subgroup might be difficult to identify with other classical histopathologic characteristics that are linked to an adverse prognosis.

**Electronic supplementary material:**

The online version of this article (10.1007/s00428-020-02764-1) contains supplementary material, which is available to authorized users.

## Introduction

“Over 1.8 million new colorectal cancer cases and 881,000 deaths are estimated to occur in 2018, accounting for about 1 in 10 cancer cases and deaths. Overall, colorectal cancer ranks third in terms of incidence but second in terms of mortality” [1]. These estimates illustrate that colorectal cancer is a serious, worldwide public health problem. Today, tumor-node-metastasis (TNM) staging of the American Joint Committee on Cancer (AJCC) is still considered the gold standard for staging. Irrespective thereof, tumor stroma ratio (TSR) is a promising new prognostic biomarker in colon cancer, which could provide additional risk stratification for therapy adaption. In colon cancer, studies were able to show that a high proportion of intratumoral stromal tissue is associated with a worse prognosis [2–7]. The prognostic value of the tumor stroma has also been shown in breast, ovarian, cervical, gastric, and esophageal cancer [8–13]. The complete biological role of stroma is not fully understood, yet. But it is known that the stroma has an important role for the supply of the tumor and the formation of blood vessels and that the stroma can promote tumorigenesis and metastasis [14–17]. The mesenchymal subtype (CMS 4) of the consensus colon cancer subtypes is associated with a worse prognosis [18]. For colon cancer, TSR assessment recommendations have been published recently [11]. Next to the assessment of stroma proportion by visual estimation, some groups used morphometric methods for the assessment of the stroma proportion [3, 5, 19, 20]. In rectal cancer, one group investigated the tumor stroma ratio with computer-aided quantification [21].

The objective of this study was the investigation of the prognostic significance of stroma/tumor proportion at different tumor sites of colon adenocarcinomas (pT3/4) of no special type with a simple semiautomatic approach with the open-source program ImageJ [22].

## Materials and methods

### Case collective

We retrospectively evaluated 215 patients with colon adenocarcinomas of no special type, pT3/4, N± , M0, R0, and at least 3-month survival after surgery. All patients underwent surgery at the University Hospital Augsburg between January 2002 and December 2011, and none of the patients had received preoperative chemo- or radiotherapy. We excluded 9 patients because of insufficient tumor tissue or insufficient immunohistochemical staining result (e.g., because of improper fixation) from further analysis. Location: we defined right-sided tumors from oral to the left colonic flexure (excluded) and left-sided tumors from the left colonic flexure (included) to aboral. The evaluation of lymphatic vessel and venous invasion were performed in each case on all tumor H&E slides. Follow-up data were provided by the Tumor Data Management of the University Hospital Augsburg and complemented with data of the patient files. Tumor budding was graded according to ITBCC, and in each case, the consensus rating was used to define the budding grade, as recently published [23, 24]. The study protocol was approved by the Institutional Review Board of University Hospital Augsburg, Germany.

### Assessment of tumor and stroma proportion

In each case, three regions were selected for the assessment of the tumor proportion (TP)/stroma proportion (SP). These were (1) FroTP, defined as the region with the deepest infiltration, measured directly at the invasive front; (2) minimal tumor proportion (MinTP), defined as the region suspected to have the highest SP/lowest TP on the slide; and (3) maximum tumor proportion (MaxTP), defined as the region suspected to have the lowest SP/highest TP. In each case, one representative H&E section (and a corresponding cytokeratin staining), which did show the deepest TNM relevant infiltration depth, was used by Be. Ba. or in difficult cases by cooperation of Be. Ba. and Be. Ma. for selection of the regions.

**Fig. 1:**
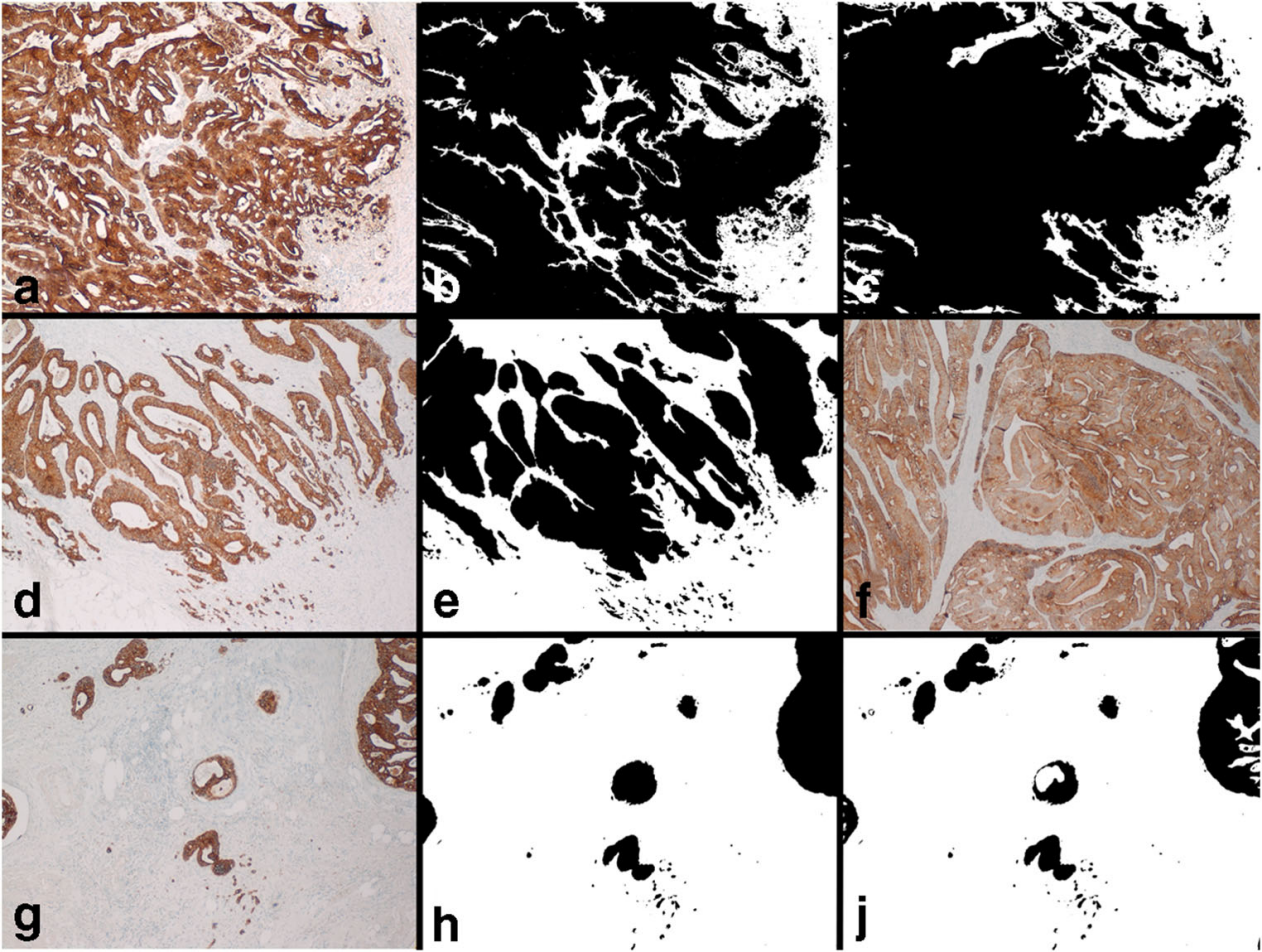
**a** CK-IHC of FroTP of a tumor with high tumor proportion. **b** Tumor A before measurement and after manual improvements (see discussion). **c** Tumor A after binary coding. **d** CK-IHC of FroTP of a tumor with medium tumor proportion. **e** Tumor D before measurement. **f** MaxTP region of Tumor D. **g** CK-IHC of FroTP of a tumor with low tumor proportion. **h** Tumor G before measurement and after manual improvements (see discussion). **j** Tumor G after binary coding

The workflow was the following: After an overview of the H&E slide, we selected the best fitting regions to the above-mentioned definitions with exclusion of areas containing significant amount of blood vessels, necrosis, abscesses, mucinous areas, or large glandular lumen. Glandular tumor lumen could not be ignored from analysis because of the subsequent digital workflow (binary coding). Lumen was assigned to tumor proportion. The TP and SP were assessed in a field of 3.58 mm^2^, but, differing from the recommendations from van Pelts et al., we used a rectangular selection (side lengths, 2.18 mm and 1.64 mm; field size, 3.58 mm^2^) instead of a classical round microscope selection due to usage of a camera [11]. We selected only regions in which tumor cells were present at all four borders of the image field. The selection process of each region was performed using a microscope (Olympus, BX43F, Tokyo, Japan) with attached camera with connection to a computer (ProgRes® Speed XTcore5 with combined software: Capture Pro 2.9.0.1). Digital images were captured (× 4 objective) of the immunohistochemical stained slide against a cytokeratin that highlighted the tumor tissue. If a histochemical stained slide, corresponding to the selected H&E slide, was already available, we used that one. If not, we prepared a cytokeratin AE1/AE3 immunostaining according to our routine protocol (antibody, cell marque™, monoclonal mouse antibody; dilution 1:500; DAB Opti View IHC Detection Kit; immunostainer, Roche Benchmark Ultra). Subsequently, the open-source image processing software ImageJ (Version 1.48 v) was used for TP and SP assessment [22]. We used the function principle that immunohistochemically marked brown dark tumor tissue can be differentiated via binary coding from the surrounding light stroma tissue (Fig. 1). After binary coding of the taken images and eliminating the lumen of the tumor cells (run (“make binary”); run (“fill holes”), Be. Ba. reviewed the result, manually improving the result if necessary (especially, filled up gaps that were not recognized by the algorithm). After calibration, the images were measured (run (analyze particles)). To exclude scoring of dust particles, we only scored particles of at least 0.00023 mm^2^ in size. We defined tumor proportion as the following: sum of all tumor areas/3.58 mm^2^. We defined stroma proportion as the following: (3.58 mm^2^- sum of all tumor areas)/3.58 mm^2^.

### Statistical analysis

All statistical analyses were performed using the Statistical Package for the Social Sciences (SPSS software version 24.0 (SPSS, Chicago, IL, USA)). The database was partitioned into an exploration and validation set for cutoff determination. The exploration set (*n* = 104) included all cases with odd study numbers of the original set (before exclusion of patients), and the validation set included all cases with even study numbers (*n* = 102) of the original set. The chi-square test was applied for comparisons of categorical data. The Mann-Whitney U test was applied for comparisons between continuous and ordinal variables between two groups. The Kruskal-Wallis test was applied for comparisons between continuous and ordinal variables for more than two groups. Univariate event/survival analyses were done according to the Kaplan-Meier method (log-rank test) for assessment of statistical significance. Cox regression analysis (forward, likelihood ratio) was performed to investigate the independence of univariate-identified risk factors. Results were considered statistically significant if *p* < 0.05. Continuous variables were demonstrated as mean ± standard deviation (SD), if not otherwise specified.

## Results

### Patients and scoring results

The mean age of the 206 patients at date of diagnosis was 70.0 ± 11.3 years, the mean follow-up time was 5.3 ± 3.5 years, and 85 (41%) patients died during the follow-up period. The detailed clinicopathological characteristics are shown in Table 1.

**Table 1:** Clinicopathological characteristics

Variable		n = 206		High TP (*n* = 42)		Medium TP (*n* = 133)		Low TP (*n* = 31)		*p* value
Mean age (years)		70.0 ± 11.3		73.9 ± 12.2		68.6 ± 11.4		70.8 ± 8.3		0.013
Mean follow-up (years)		5.3 ± 3.5		4.8 ± 3.5		5.7 ± 3.6		4.4 ± 2.7		0.139
Mean lymph node harvest (*n*)		23 ± 13		19 ± 8		24 ± 14		26 ± 15		0.084
Positive lymph nodes (*n*)		1.2 ± 2.3		0.9 ± 1.9		1.1 ± 2.3		1.7 ± 2.4		0.485
Sex										0.934
	Female	88	43%	17	40%	58	44%	13	42%	
	Male	118	57%	25	60%	75	56%	18	58%	
T status										0.988
	pT3	180	87%	37	88%	116	87%	27	87%	
	pT4	26	13%	5	12%	17	13%	4	13%	
Nodal status										0.742
	Negative	128	62%	28	67%	82	62%	18	58%	
	Positive	78	38%	14	33%	51	38%	13	42%	
Vascular invasion										0.587
	Negative	186	90%	39	93%	118	89%	29	94%	
	Positive	20	10%	3	7%	15	11%	2	6%	
Lymphovascular invasion										0.061
	Negative	171	83%	40	95%	106	80%	25	81%	
	Positive	35	17%	2	5%	27	20%	6	19%	
Grading										0.394
	Low grade	141	68%	29	69%	94	71%	18	58%	
	High grade	65	32%	13	31%	39	29%	13	42%	
Tumor budding										0.029
	Bd 1	168	82%	38	90%	109	82%	21	68%	
	Bd 2	25	12%	3	7%	18	14%	4	13%	
	Bd 3	13	6%	1	2%	6	5%	6	19%	
Location										0.999
	Right	127	62%	26	62%	82	62%	19	61%	
	Left	79	38%	16	38%	51	38%	12	39%	
Microsatellite status										0.132
	MSS	180	87%	38	90%	112	84%	30	97%	
	MSI	26	13%	4	10%	21	16%	1	3%	
Distant metastasis										0.003
	No	165	80%	28	67%	116	87%	21	68%	
	Yes	41	20%	14	33%	17	13%	10	32%	
Death										0.003
	No	121	59%	16	38%	89	67%	16	52%	
	Death	85	41%	26	62%	44	33%	15	48%	
5-year survival (*n* = 160)										0.042
	Survived	97	61%	18	49%	67	68%	12	48%	
	Death	63	39%	19	51%	31	32%	13	52%	

*p* values are shown for difference between the low, medium, and high tumor proportion group, classified by cutoffs at FroTP

*MSI* microsatellite instable, *MSS* microsatellite stable

The mean/median TP was 36%/36% at FroTP, 32%/31% at MinTP, and 71%/73% at MaxTP, respectively (Table 2). The MinTP was assumed to be in the same region as the FroTP in 161 tumors (78%).

**Table 2:** Overview of the tumor proportion in the different regions

Variable	All (*n* = 206)	High TP (*n* = 42)	Medium TP (*n* = 133)	Low TP (*n* = 31)	*p* value
Mean FroTP	36 ± 18	62 ± 7	34 ± 11	11 ± 8	< 0.001
Mean MinTP	32 ± 16	49 ± 17	32 ± 10	11 ± 3	< 0.001
Mean MaxTP	71 ± 13	77 ± 9	70 ± 13	65 ± 15	0.001

*p* values are shown for difference between the low, medium, and high tumor proportion group, classified by cutoffs at FroTP

### Determination of cutoff values

**Fig. 2:**
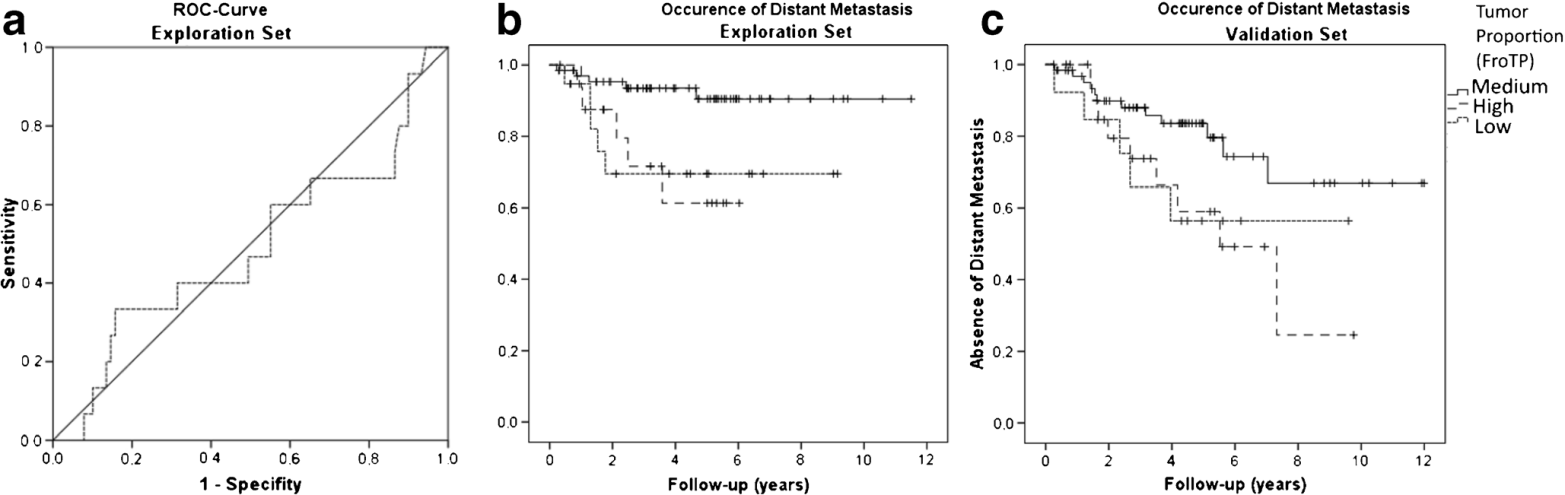
**a** ROC curve for the occurrence of distant metastasis in the exploration set. **b** Kaplan-Meier curves for the occurrence of distant metastasis in the exploration set (high versus medium tumor proportion *p* = 0.015; low versus medium tumor proportion *p* = 0.009). **c** Kaplan-Meier curves for the occurrence of distant metastasis in the validation set (high versus medium tumor proportion *p* = 0.037; low versus medium tumor proportion *p* = 0.105)

The stroma high group included 76% (FroTP) and 86% (MinTP) of the cases according to a cutoff at 50%. As the tumor proportion in our study, and consequently the corresponding group sizes, differed considerably (if the classical cutoff was applied) from published literature (discussed later), we divided our population into an exploration and validation set for cutoff determination. We used a receiver operating characteristic (ROC) curve approach to determine the optimal cutoff point for the prediction of distant metastasis (Fig. 2a, ROC curve exploration set). As the ROC curve of the exploration set of FroTP has two contrary deviations from the diagonal, we determined two cutoff values. We determined the cutoffs that resulted in the lowest *p* value in the exploration set according to the occurrence of distant metastasis by testing and adjusting. Additionally, we performed subsequently ROC analyses of MinTP and MaxTP. The analysis of MaxTP did not reveal a relevant prognostic result (ROC – curve MaxTP in supplement (S1)), and the analysis of MinTP was inferior to the analysis of FroTP (ROC – curve MinTP in supplement (S2)) but showed similar results according to the values of the cutoffs at FroTP.

### Prognostic analyses

The established cutoff values at FroTP are low TP ≤ 15% TP; medium TP, 15% < TP < 54%; and high TP ≥ 54% TP. According to these thresholds, 42 tumors (20%) were classified as high TP, 31 tumors (15%) were classified as low TP, and 133 tumors (65%) were classified as medium TP (FroTP). An overview of the mean tumor proportions in the different groups is given in Table 2. In the exploration set, a low TP as well as a high TP was associated with occurrence of distant metastasis in comparison to medium TP (*p* = 0.009 and *p* = 0.015, Fig. 2b). In the validation set, a high TP was also associated with occurrence of distant metastasis in comparison to medium TP (*p* = 0.037, Fig. 2c). The association of a low TP to the occurrence of distant metastasis could not be confirmed, although the *p* value did show a trend towards significance in comparison to the medium TP group (*p* = 0.105) (Fig. 2c).

**Fig. 3:**
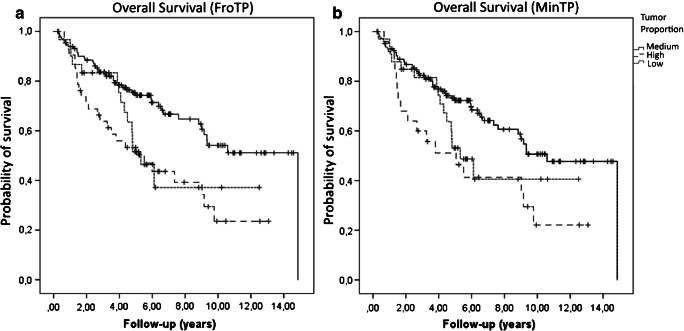
**a** Kaplan-Meier curves for the overall survival based on the overall patient population and the tumor proportion in the front region (FroTP) (high versus medium tumor proportion *p* = 0.001; low versus medium tumor proportion *p* = 0.03). **b** Kaplan-Meier curves for the overall survival based on the overall patient population and the tumor proportion in the region with the lowest estimated tumor proportion (MinTP) (high versus medium tumor proportion *p* = 0.006; low versus medium tumor proportion *p* = 0.094)

A further Kaplan-Meier analysis in the overall patient population showed a significant adverse overall survival for patients with a high and low TP in comparison to patients with medium TP (*p* = 0.001 high TP; p = 0.03 low TP, Fig. 3a). The 5-year survival rate was 49% in patients with high TP, 48% in patients with low TP, and 68% in patients with medium TP (*p* = 0.042, *n* = 160).

We applied the established cutoffs at FroTP region also at the MinTP region. Thereby, in the overall population (*n* = 206), 147 patients (71%) were assigned to medium TP group, 25 patients (12%) to the high TP group, and 34 patients (17%) to the low TP group. A high TP was associated with occurrence of distant metastasis and worse overall survival in comparison to the medium TP group (*p* = 0.021 and *p* = 0.006). A low TP was associated with occurrence of distant metastasis, and the *p* value did show a trend towards significance according to an adverse overall survival in comparison to the medium TP group (*p* = 0.008 and *p* = 0.094, Fig. 3b).

### Multivariate Cox regression

Additionally, we performed a multivariate Cox regression (forward, likelihood ratio) for the occurrence of distant metastasis including the following risk factors (all variables with a significance level *p* ≤ 0.1 in univariate analyses): age (≥ 70 years versus < 70 years), pT, pN (positive versus negative), lymphovascular invasion, sidedness, microsatellite status, tumor budding, FroTP (defined as categorical variable), and MinTP (defined as categorical variable). T status, microsatellite status, tumor budding, as well as FroTP were integrated into the model as independent risk factors. The hazard ratios for the low and high TP groups (FroTP) were 2.7 (1.2–6.0, *p* = 0.016) and 3.2 (1.6–6.7, *p* = 0.001) (Table 3). Furthermore, we performed another multivariate Cox regression (forward, likelihood ratio) for overall survival including factors with a significance level *p* ≤ 0.1 in univariate analyses. We excluded distant metastasis as risk factor from this analysis, as it indicates tumor progression during follow-up. In this model, the FroTP, as well as the T status, and the age were independent risk factors (Table 3).

**Table 3:** *p* values and hazard ratios for univariate and multivariate analyses

	Univariate analyses			Multivariate Cox regression analyses			
Variable	Distant metastasis *n* = 206	Overall survival *n* = 206	5-year survival *n* = 160	Distant metastasis, hazard ratio, and 95% CI	P value	Overall survival hazard ratio, and 95% CI	*p* value
Sex	0.18	0.66	0.639				
Mean age (years)	0.096	< 0.001	0.002		0.17		
> = 70 years						2.2 (1.4–3.6)	0.001
< 70 years							
Mean lymph node harvest	0.14	0.24	0.573				
> = 23 lymph nodes							
< 23 lymph nodes							
T status	< 0.001	0.018	0.012				
T3							
T4				4.7 (2.3–9.7)	< 0.001	2.2 (1.2–3.9)	0.01
Nodal status (+ vs. -)	0.06	0.25	0.468		0.69		
Vascular invasion	0.19	0.49	0.64				
Lymphovascular invasion	0.04	0.39	0.133		0.14		
Grading	0.34	0.89	0.697				
Tumor budding	0.02	0.34	0.179	1.6 (1.003–2.4)	0.048		0.23
Sidedness	0.047	0.669	0.598		0.071		
Microsatellite status	0.03	0.23	0.374				
MSS							0.14
MSI				0.12 (0.02–0.92)	0.04		
Tumor proportion (FroTP)	0.001	0.002	0.042				
High tumor proportion				3.2 (1.6–6.7)	0.001	2.1 (1.3–3.4)	0.004
Medium tumor proportion							
Low tumor proportion				2.7 (1.2–6.0)	0.016	2.0 (1.1–3.7)	0.02
Tumor proportion (MinTP)	0.009	0.01	0.084		ns		ns
Distant metastasis		< 0.001	< 0.001				

*CI* confidence interval, *MSI* microsatellite instable, *MSS* microsatellite stable, *ns* not significant

*FroTP* region at invasion front with deepest infiltration; *MinTP* region with suspected lowest tumor proportion

### Characteristics of the different groups

There were significant differences between the three groups according to the frequency of tumor budding and the age of the patients (see Table 1). A low TP was associated to tumor budding (*p* = 0.012 low TP vs. high TP; *p* = 0.046 low TP vs. medium TP) and a higher lymph node harvest (*p* = 0.042 low TP vs. high TP; *p* = 0.480 for low TP vs. medium TP). A high TP was associated to the absence of tumor budding (*p* = 0.012 high TP vs. low TP; *p* = 0.192 high TP vs. medium TP), lymphovascular invasion (*p* = 0.049 high TP vs. low TP; *p* = 0.018 high TP vs. medium TP), and a lower lymph node harvest (*p* = 0.042 high TP vs. low TP; *p* = 0.057 high TP vs. medium TP).

## Discussion

In this study, we investigated the tumor and stroma proportion in different regions of 206 pT3 and pT4 adenocarcinomas (NOS) of the colon in a semiautomatic image analysis approach. We used the open-source software ImageJ and the principle that immunohistochemically marked tissue can be differentiated via binary coding from surrounding light stroma tissue. We divided our patient population into an exploration and validation set for cutoff determination. In the validation set, we could confirm that a high TP/low SP (≥ 54% TP *p* = 0.037) is associated with occurrence of distant metastasis. In the overall population, a high TP at FroTP was significantly associated with a worse overall survival and lower 5-year survival rate (see Fig. 3 and Table 1). Additionally, a multivariate Cox regression did show that a high TP is an independent risk factor for occurrence of distant metastasis beside the T status, microsatellite status, and the tumor budding grade. The hazard ratio of patients with high TP was 3.2 (1.6–6.7) in comparison to medium TP (*p* = 0.001). These findings are quite surprising, because, so far, only a low TP/high SP has been linked to a worse prognosis [14]. In this study, a low TP/high SP (≤ 15% TP) at FroTP was also associated with a worse overall survival (*p* = 0.03 for low TP vs medium TP, Fig. 3), and the 5-year survival rate was lower than in the medium TP group (*p* = 0.042). The results of the prognostic impact of the MinTP region seem to be similar but all in all a bit inferior than at FroTP. We could not find a relevant prognostic impact of the MaxTP region (ROC curve supplement), which emphasizes the relevance of the selected region for the assessment. The different mean TPs in the MinTP (32%), FroTP (36%), and MaxTP (71%) show the extent of tumor stroma heterogeneity. In literature, different tumor regions have been investigated according to their prognostic value. Van Pelt et al. recommend that the region with the highest stroma proportion on the slide of the most invasive part should be decisive [11]. Besides that, other groups could show that a high stroma content of the “whole” tumor, as well as at the luminal surface, is prognostic [5, 20]. The luminal region has the advantage that biopsies could be scored, but all in all, the recommendations of van Pelt et al. seem to be the most promising for a near-term implementation as an easy diagnostic tool [25].

Interestingly, high TP tumors miss conventional adverse histological features. These tumors had a low proportion of tumor budding (*p* = 0.012 for low TP and *p* = 0.192 for medium TP) and a low proportion of lymphovascular invasion (*p* = 0.049 for low TP and *p* = 0.018 for medium TP). Furthermore, less lymph nodes have been harvested in these tumors (*p* = 0.042 for low TP and *p* = 0.057 for medium TP). Assuming that the lymph node count is a surrogate indicator of the immunological response to the tumor, one might speculate that the immunological landscape plays an important role in these cases [26]. Further studies could clarify this relation more closely and potentially identify therapeutic opportunities for this subgroup. Patients with a high TP were significantly older than the rest of the patients (*p* = 0.004 for medium TP and *p* = 0.031 for low TP), but the Cox regression analysis did show that the adverse prognostic effects are independent of age. Other significant differences could not be observed (see Table 1). A morphologic reevaluation of the high TP tumors revealed no morphologic characteristics.

There are several critical aspects, which have to be taken into account in interpreting the data of this study. Despite highly significant results, one has to keep in mind that the data is from a single center and the design is retrospective with all the well-known associated drawbacks.

The cutoff determination for TP, for defining this subgroup, has been determined in an exploration and validation set for the occurrence of distant metastasis; however, the survival analyses have been performed in the overall patient population, which consists of the two sets. The presented approach of TP and SP assessment based on binary coding with ImageJ has advantages but also limitations. The presented workflow is not as time and cost-effective as the conventional assessment on H&E. However, the necessary input and resources are limited, as the workflow can be established with basic equipment and only requires an open-source software. After binary coding, the result has to be manually reviewed and if necessary reworked. Two typical causes for rework can be seen in Fig. 1c and j. In Fig. 1j, the binary coding and the “fill the holes” algorithm missed to fill up all holes. Therefore, all lumen had to be stained manually black (Fig. 1h). Comparing Fig. 1b and c shows that a central non-tumor part has been falsely stained black. After drawing a small white connection to the surrounding light stroma area, the area could be correctly recognized (Fig. 1b). Of course, this kind of review and reworking process is a source of interobserver variability, but we consider that of lesser importance because the anti-cytokeratin staining can be used as model. All in all, it has been reported that the reproducibility of TSR scoring is good [11]. Irrespective thereof, we assume that the direct comparability of our results to studies in the existing literature is limited. In comparison to West et al., the median TP was considerably lower (22/26 percentage points in comparison to FroTP/MinTP), and after applying the established cutoff at 50% stroma, a high proportion of patients has been assigned to the high stroma group (76% for FroTP/86% for MinTP) [5]. In most studies, the high SP group included only 20–30% of all tumors [3, 4, 19]. We assume the main reason for the lower TP and higher SP is that we have used an anti-cytokeratin stained slide for the selection of the regions and that we accepted also tumor buds, as part of the tumor, at the borders of the region (Fig. 1). A further factor that influenced the TP/SP is that we were not able to ignore different areas from scoring. Glandular lumen was assessed to the tumor. Therefore, in this study, the TP corresponds to the area that is covered and enclosed by tumor. Small areas with necrotic tissue, smooth muscle tissue, etc., which had to be accepted in some cases after carefully taking all relevant factors (tumor tissue at all four part at the edges, overall appearance of the tumor, etc.) into account, were assigned to stroma proportion. Another reason which might have contributed to the difference in cutoffs in comparison to the literature is that semiquantitative visual estimation appears to underestimate the TSR in high stroma regions [27]. A limiting factor according to the evaluation of lymphatic and vascular invasion is that no mandatory elastica staining was performed.

## Conclusion

This study demonstrates the feasibility of a semiautomatic image analysis approach for TSR assessment in colon cancer and in accordance with literature, and the results confirm that a high stroma proportion is associated with an adverse prognosis. Beyond that, the results provide first evidence that a low stroma proportion/high tumor proportion might also be an independent risk factor for the occurrence of distant metastasis and a worse overall survival. Furthermore, the results indicate that this subgroup also differs according to the clinicopathological characteristics from other tumors.

## Electronic supplementary material

ESM 1 (PDF 280 kb)
