# Supplementary material for: Tumor proportion in colon cancer: results from a semiautomatic image analysis approach
Source: Virchows Arch. 2020 Feb 19;477(2):185–93. doi: 10.1007/s00428-020-02764-1 (PMC7985049; doi:10.1007/s00428-020-02764-1)
Supplement: Supplementary file 1 — (PDF 280 kb) [file 428_2020_2764_MOESM1_ESM.pdf]

Tumor Proportion in Colon Cancer – Results from a Semiautomatic Image Analysis Approach.

Benedikt Martin[1], Bettina Monika Banner[1], Eva-Maria Schäfer[1], Patrick Mayr[1, 2], Matthias Anthuber[3], Gerhard Schenkirsch[4], Bruno Märkl[1]

1 Institute of Pathology and Molecular Diagnostics, University Hospital Augsburg, Augsburg, Germany

2 Department of Radiooncology, University Hospital Augsburg, Augsburg, Germany

3 Department of Visceral Surgery, University Hospital Augsburg, Augsburg, Germany

4 Tumor Data Management, University Hospital Augsburg, Augsburg, Germany

Supplement

S1 ROC Curve TPMax according to the occurrence of distant metastasis.

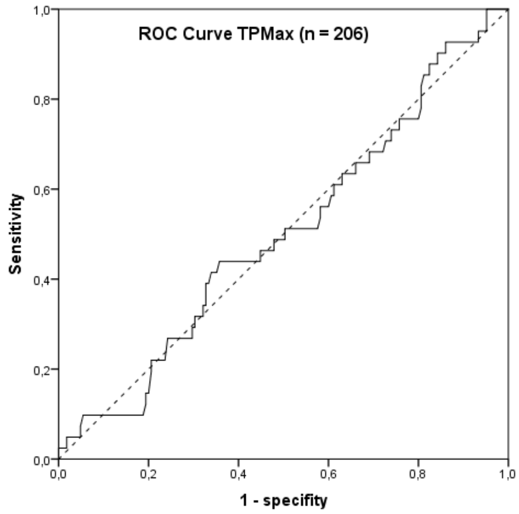

S2 ROC Curve TPMin according to the occurrence of distant metastasis.

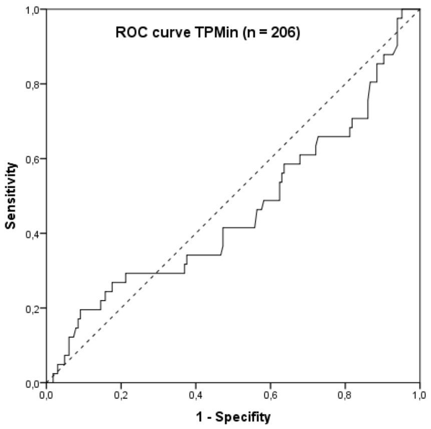

S3 Comparison of exploration and validation set.

Supplement, table 4. Comparison of exploration and validation set

Variable		All n = 206		Exploration Set n = 104		Validation Set n = 102		P-value
Mean Age (years)		70.0 ± 11.3		70.3 ± 11.8		69.9 ± 10.9		0.506
Mean Follow-up (years)		5.3 ± 3.5		5.1 ± 3.3		5.5 ± 3.7		0.728
Mean FroTP		35.9 ± 17.9		35.7 ± 17.4		36.2 ± 18.7		0.953
Mean MinTP		32.3 ± 15.9		32.7 ± 15.4		32.0 ± 16.6		0.567
Mean MaxTP		71.0 ± 13.1		70.7 ± 12.5		71.4 ± 13.9		0.451
Lymphnode Harvest (n)		23.3 ± 13.2		23.4 ± 12.7		23.3 ± 13.8		0.829
Positive Lymphnodes (n)		1.2 ± 2.3		1.2 ± 2.6		1.1 ± 1.9		0.919
Sex								0.494
	female	88	43%	42	40%	46	45%	
	male	118	57%	62	60%	56	55%	
T status								0.372
	T3	180	87%	93	89%	87	85%	
	T4	26	13%	11	11%	15	15%	
Nodal Status								0.858
	negative	128	62%	64	62%	64	63%	
	positive	78	38%	40	39%	38	37%	
Blood Vessel Invasion								0.145
	negative	186	90%	97	93%	89	87%	
	positive	20	10%	7	7%	13	13%	
Lymphovascular Invasion								0.535
	negative	171	83%	88	85%	83	81%	
	positive	35	17%	16	15%	19	19%	
Grading								0.149
	low grade	141	68%	76	73%	65	64%	
	high grade	65	32%	28	27%	37	36%	
Tumor Budding								0.301
	Bd 1	168	82%	88	84%	80	78%	
	Bd 2	25	12%	9	9%	16	16%	
	Bd 3	13	6%	7	7%	6	6%	
Location								0.266
	right-sided	127	62%	68	65%	59	58%	
	left-sided	79	38%	36	35%	43	42%	

MSI								0.104
	MSS	180	87%	87	84%	93	91%	
	MSI	26	13%	17	16%	9	9%	
Distant Metastasis								0.047
	no	165	80%	89	86%	76	75%	
	yes	41	20%	15	14%	26	26%	
Death								0.758
	no	121	59%	60	58%	61	60%	
	death	85	41%	44	42%	41	40%	
Five Year Survival				n = 84		n = 76		0.728
(n = 160)	survived	97	61%	52	62%	45	59%	
	death	63	39%	32	38%	31	41%	

Legend: p-values are shown for the comparison of the exploration and validation set

Abbreviations: MSI: microsatellite instable, mss: microsatellite stabel
